# Arsenic and Quinine in Intermittent

**Published:** 1865-02

**Authors:** 


*Arsenic and Quinine in Intermittent.*—Lecturing on the subject of intermittent fever, Dr. Ward, Physician to the Dreadnought Hospital, says: "I have fairly tried the liquor potassæ arsenitis against the quinine, in doses as full and frequently repeated as I dared to venture upon, and have continued it until irritation of the intestinal mucous coat rendered desistance imperative; but have not succeeded in checking the paroxysms as with quinine.—Some physicians, admitting the greater efficacy of quinine in arresting the paroxysms, have claimed for arsenic, and also for strychnia, greater power in preventing a relapse. I believe, however, that quinine is quite effective for this purpose if continued in small doses for a considerable time, and also that it is the best prophylactic against the disease. A captain, who consulted me a year or two back, said that he was on the point of sailing for Jamaica, and that had never been there without contracting sever ague. I recommended him to take quinine, in small doses, two or three times a day, all the way out. He did so; and on his return to England reported that for the first time he had enjoyed immunity from the disease.

